# An Adaptive Image Enhancement Technique by Combining Cuckoo Search and Particle Swarm Optimization Algorithm

**DOI:** 10.1155/2015/825398

**Published:** 2015-02-15

**Authors:** Zhiwei Ye, Mingwei Wang, Zhengbing Hu, Wei Liu

**Affiliations:** ^1^School of Computer Science, Hubei University of Technology, Wuhan 430068, China; ^2^School of Educational Information Technology, Huazhong Normal University, Wuhan 430070, China

## Abstract

Image enhancement is an important procedure of image processing and analysis. This paper presents a new technique using a modified measure and blending of cuckoo search and particle swarm optimization (CS-PSO) for low contrast images to enhance image adaptively. In this way, contrast enhancement is obtained by global transformation of the input intensities; it employs incomplete Beta function as the transformation function and a novel criterion for measuring image quality considering three factors which are threshold, entropy value, and gray-level probability density of the image. The enhancement process is a nonlinear optimization problem with several constraints. CS-PSO is utilized to maximize the objective fitness criterion in order to enhance the contrast and detail in an image by adapting the parameters of a novel extension to a local enhancement technique. The performance of the proposed method has been compared with other existing techniques such as linear contrast stretching, histogram equalization, and evolutionary computing based image enhancement methods like backtracking search algorithm, differential search algorithm, genetic algorithm, and particle swarm optimization in terms of processing time and image quality. Experimental results demonstrate that the proposed method is robust and adaptive and exhibits the better performance than other methods involved in the paper.

## 1. Introduction

The visual quality of the most recorded images is often brought down in the course of digital image acquisition because of some factors such as uneven illumination, the noise produced in the transmission, and D/A transformation. As a consequence, image quality usually needs to be improved before image analysis; image enhancement is an elementary step in digital image processing and analysis [1]. The goal of enhancement techniques is to process an image so that the result is more suitable than the original image for specific applications or set of objectives. Many methods based on gray-level histogram modification, the local contrast transformation, edge analysis, and the “global” entropy transformation have been proposed to enhance image. In [2], Cheng and so forth defined a new approach to fuzzy entropy and used it to automatically select the fuzzy region of membership function so that an image was able to be transformed into fuzzy domain with maximum fuzzy entropy. Tang and so forth put forward an image enhancement algorithm for images compressed using the JPEG standard [3]. In general, the enhancement methods may be summarized as two main categories: frequency domain and spatial domain. Frequency domain processing techniques are based on modifying the Fourier transform of an image. Spatial domain refers to the image plane itself, and approaches in this category are based on direct manipulation of pixel in an image. In the paper, our work is based on spatial domain.

Histogram transformation is one of the most basic techniques for spatial domain enhancement of gray-level images [4]. As the adjustment of histogram, the structural relationship of intensity values of the pixel will be changed. The expression of histogram is a discrete probability density function about gray-levels and reflects the relative frequency distribution of gray-levels. Histogram equalization is a method in image processing of contrast adjustment by increasing the global contrast of the image, especially when the usable data of the image is represented by close contrast values [5]. However, the intensity values of pixel will be adjusted to an average position, which cannot highlight the details of the original image. As the basis for numerous spatial domain techniques of image contrast enhancement, it usually manipulates the histogram by the transformation function to obtain the required target. Consequently, this method is to deliver the maximum information contained in the original image. Linear contrast stretching is to increase the dynamic range of the gray-levels in the original image by using a linear transformation [6]. It mainly enhances the contrast grade of the original image, but the threshold must be manually set. If the threshold is not suitable, the enhanced image may be worse than original image. Due to the absence of general standard of the image quality, which could be served as a design criteria for image enhancement algorithms, improving image contrast is difficult by simply stretching the histogram of the image or using simple gray-level transformations [7]. Therefore, recently some novel methods using evolutionary computation and metaheuristic optimization algorithms based on some quality measures of image enhancement have been put forward to further deal with the problem of image enhancement.

Evolutionary computation is a subfield of artificial intelligence that involves continuous optimization and combinatorial optimization problems [8–10]. In [8], Civicioglu introduced a new evolutionary algorithm named backtracking search algorithm (BSA) for solving real-valued numerical optimization problems. In [9], a new evolutionary algorithm called differential search algorithm (DSA) is presented to solve the problem of transforming the geocentric Cartesian coordinates into geodetic coordinates. In [10], Hrelja et al. used particle swarm optimization (PSO) to propose the modelling of turning process. In particular, some classical evolutionary computation algorithms have been previously used to perform image enhancement [11–14]. In [11], Saitoh proposed a method to enhance the contrast of a gray-level image using genetic algorithm (GA) that measures the fitness of an individual by evaluating the intensity of spatial edges included in the image. Gorai and Ghosh have applied an objective criterion for measuring image enhancement which considers entropy and edge information of the image with the help of PSO in [12]. Coelho and so forth presented three differential evolution approaches based on chaotic sequences using logistic equation for image enhancement process in [13]. In [14], the different transformation functions with different parameters were used to produce the enhanced image by GA. These methods have obtained fair good results on image enhancement. However, the definition of image quality measure is imperfect; there are few of objective functions which are able to make a good versatility for all images. Moreover, GA and PSO are easy to fall into local optima. Recently, hybridization of metaheuristics has received great interest. In the paper, a novel image enhancement technique using a modified quality measure and blending of cuckoo search and particle swarm optimization algorithm is proposed.

Cuckoo search (CS) algorithm is a population-based stochastic global search algorithm [15]. In [16], the authors have made a conceptual comparison of cuckoo search (CS), particle swarm optimization (PSO), and genetic algorithm (GA). The final results indicate that CS can better converge to the optimal solution, but its convergence rate is not very well. A hybrid approach of cuckoo search (CS) and particle swarm optimization (PSO) is presented and experimental results demonstrate that the hybrid method (CS-PSO) is a better method compared with other simplex evolutionary algorithm in [17]. Hence, in this paper, on the basis of the newly modified image quality criteria, CS-PSO is employed to perform gray-level image contrast enhancement. We compare the new method with the previously presented methods. The experimental results demonstrate that our method outperforms the other ones from the subjective and objective viewpoints.

The remainder of the paper is organized as follows: In Section 2, the proposed contrast enhancement mechanism and related function used are given. In Section 3, basis theory of CS-PSO (original CS algorithm, CS-PSO algorithm) is illustrated in brief. In Section 4, the proposed enhancing model is detailed. In Section 5, simulation results and discussion are displayed. Finally, Section 6 draws a conclusion.

## 2. Proposed Contrast Enhancement Mechanism and Related Function

The simplest way to carry out contrast enhancement is global intensity transformation. In the way, by utilizing lookup tables, the intensity levels in an image are mapped into a new set of grey levels thus changing the image parameters like the contrast [18]. The main objective in global intensity transformation is to obtain a lookup table or transfer function which yields an output image with improvement in desired parameters. For image enhancement, a transformation function is required which will map the intensity value of each pixel from the input image into a new intensity value for the corresponding pixel to produce the enhanced image. To evaluate the quality of the enhanced image and acquire the optimal enhancement parameters automatically, an objective function is needed. In this section, we will discuss the related function used for the proposed work.

### 2.1. Transformation Function

In general, image enhancement is done on spatial domain by using a transformation function which produces a new intensity for each pixel of the original image to generate the enhanced image. If the spatial relationship of pixel values is changed, the enhanced image will be altered along with it. As the image size increases, the time complexity of the algorithm will increase hugely. Tubbs proposed a fitting transformation function method by using incomplete Beta function, which does not have to know the spatial distribution of the original image [19]. In [20], incomplete Beta function enhancement method based on PSO is applied to aerial and satellite remote sensing image enhancement. The incomplete Beta function is defined as follows:

(1) $$\begin{array}{c} F\left(u\right)=B^{-1}\left(\alpha ,\beta \right)*\overset{u}{\underset{0}{\int }}t^{\alpha -1}{\left(1-t\right)}^{\beta -1}dt \end{array}$$

(2) $$\begin{array}{c} B\left(\alpha ,\beta \right)=\overset{1}{\underset{0}{\int }}t^{\alpha -1}{\left(1-t\right)}^{\beta -1}dt. \end{array}$$

**Figure 1:**
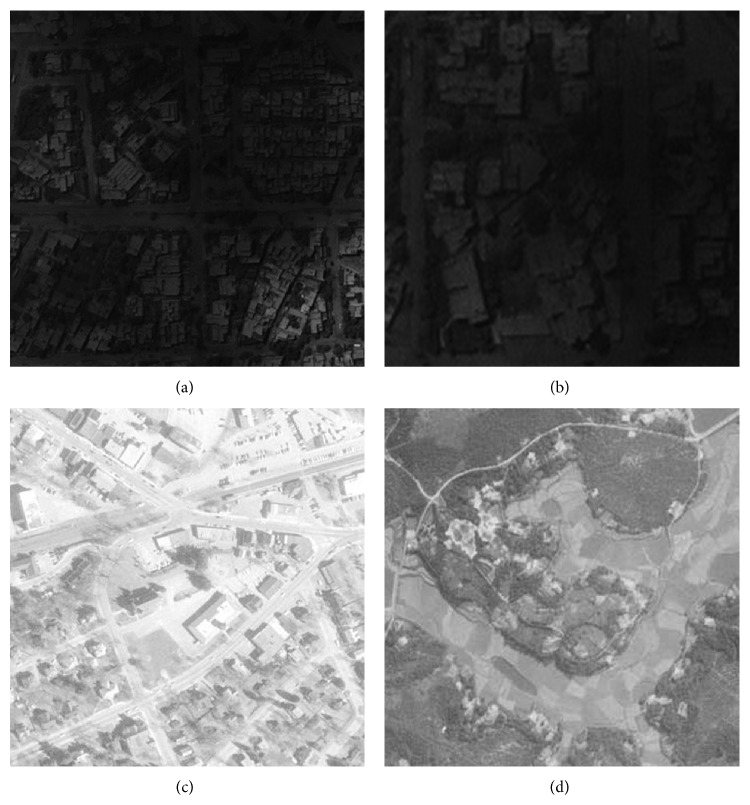
Original images: (a) I1, (b) I2, (c) I3, and (d) I4.

In (1) and (2), *B*(*α*, *β*) is the Beta function, *t* is the variable of integration, *u* is the gray-levels after normalization of the original image (Figure 1), and two parameters are introduced in the incomplete Beta function, namely, *α* and *β* are to obtain as large fitness value as possible in the enhanced image.

### 2.2. Objective Function

To evaluate the quality of an enhanced image without human intervention, an objective function is needed which is able to estimate the image quality impartially as far as possible. Some objective functions have been given in [12, 21, 22].

In [12], the authors proposed an objective function which was formed by combining three performance measures, namely, entropy value, sum of edge intensities, and number of edges. Compared with the original image, the enhanced image has more number of edges and enhanced version should have a higher intensity of the edges. This function can make a fair good evaluation for enhanced image, but its convergence rate is unsatisfactory, which is defined as

(3) $$\begin{array}{c} F=\log\left(\log\left(E\left(I_{s}\right)\right)\right)\times \frac{n\_\text{edgels}\left(I_{s}\right)}{M\times N}\times H\left(I_{e}\right), \end{array}$$

where *M* and *N*, respectively, denote the number of columns and rows of the original image. *E*(*I*_*s*_) stands for the sum of *M* × *N* pixel intensities of Sobel edge image. *n*_edgels is the number of pixels, whose intensity value is above a threshold in the Sobel edge image. *H*(*I*_*e*_) is the entropy value of the enhanced image.

In [21], an objective function formed by using the statistical variable of enhanced image was presented. The function was independent of viewing conditions and individual observers, but it could only be applied to a fraction of test images, which is given in

(4) $$\begin{array}{c} Q=\frac{4\sigma_{xy}\overset{-}{x}\;\overset{-}{y}}{\left(\sigma_{x}^{2}+\sigma_{y}^{2}\right)\left[{\left(\overset{-}{x}\right)}^{2}+{\left(\overset{-}{y}\right)}^{2}\right]}, \end{array}$$

where $\overset{-}{x}$ and *σ*_*x*_, respectively, indicate mean value and variance of the intensity values for original image. $\overset{-}{y}$ and *σ*_*y*_, respectively, express mean value and variance of the intensity values for enhanced image. *σ*_*xy*_ represents covariance of the intensity values between original image and enhanced image.

In [22], the authors proposed an objective function which only uses the intensity values of pixel. It can promptly obtain the optimal solution, but this function cannot show the correlation of adjacent pixel; it is not fully suitable for solving the problem of image enhancement, which is as in

(5) $$\begin{array}{c} F=\frac{1}{M\times N}\overset{M}{\underset{x=1}{\sum }}\;\overset{N}{\underset{y=1}{\sum }}I^{2}\left(x,y\right)-{\left[\frac{1}{M\times N}\overset{M}{\underset{x=1}{\sum }}\;\overset{N}{\underset{y=1}{\sum }}I\left(x,y\right)\right]}^{2}, \end{array}$$

where *M* and *N*, respectively, denote the number of columns and rows of the original image and *I*(*x*, *y*) indicates the intensity value of each pixel.

In the theory of signal processing, entropy value reveals the information content in the image. It is widely used in determining the evaluation criterion in image processing. Histogram reflects a discrete probability density function about gray-levels and reports the relative frequency distribution of gray-levels. Hence, in the paper a novel objective function is proposed as

(6) $$\begin{array}{c} F\left(I_{e}\right)=\log\left(\frac{E\left(I_{e}\right)*N_{T}}{\Delta h}\right)*\left(\frac{\text{sum}\left(h_{\text{Th}}\right)}{\left(M*N\right)}\right), \end{array}$$

where *M* denotes the number of columns and *N* denotes the number of rows of the original image. Based on the histogram of the enhanced image *I*_*e*_, *h*_*i*_ is the probability of occurrence *i*th gray value and Δ*h* is the variance of *h*_*i*_. *N*_*T*_ is the number of gray-levels in which the probability density is greater than a predetermined threshold value *T*. sum(*h*_Th_) is the number of probability density in which the gray-levels are within the range of another predetermined threshold value Th. *E*(*I*_*e*_) is the entropy value which is calculated on the enhanced image *I*_*e*_ as follows:

(7) $$\begin{array}{c} E\left(I_{e}\right)=-\overset{255}{\underset{i=0}{\sum }}e_{i}\\ e_{i}=\left\{\begin{array}{cc} h_{i}\text{lo}{\text{g}}_{2}\left(h_{i}\right) & \text{while}\;\;h_{i}\neq 0\\ 0 & \text{otherwise}. \end{array}\right. \end{array}$$

## 3. The Basic Theory of CS-PSO 

### 3.1. Original CS Algorithm

Cuckoo search (CS) is an evolutionary algorithm proposed by Yang and Deb in 2009 [15]. This evolutionary algorithm is a search strategy model on brood parasitism of some cuckoo species by laying their eggs in the nests of other host birds. If a host bird discovers the eggs are not its own, it will either fling these alien eggs or simply desert its nest and put up a new nest elsewhere. In a CS system, each cuckoo species alter their position as time goes, and every egg in the nest stands for only one new solution. The better new solution will take place of the solution which is relatively worse in the nest. For simplicity, only three idealized rules are utilized to describe the CS algorithm as follows [15, 23].

1. Each cuckoo lays one egg at a time and dumps it in a randomly selected nest.
2. The best nests with high quality of eggs (solutions) will be kept up to the next generation.
3. The number of available host nests is fixed, and a host can discover an alien egg with a probability *P*_*a*_ ∈ [0,1]. In this case, the host bird can either throw the egg away or abandon the nest so as to build a completely new nest in a new location.

Moreover, a mass of studies have indicated that flight behaviors of many animals and insects have the typical characteristics of the Levy flights [24]. In view of these breeding and flight behaviors, the authors in [15] presented the CS algorithm.

For an optimization problem, the quality of a solution could simply be corresponding to the fitness value of the objective function. Other forms of fitness can be defined in a parallel way to the objective function in other evolutionary algorithms. Three rules are defined in the algorithm; first, each egg in a nest stands for a solution; second, a cuckoo egg denotes a new solution; third all of the cuckoos are evaluated by the fitness value of the objective function to be optimized and have velocities which directly decide the cuckoos' flying; the intent is to use the new better solutions to replace the not-so good solution in the nests.

In view of these three rules, the primary steps of the CS can be described with the pseudocode in Pseudocode 1.

In order to generate the new solutions *x* ^(*t* + 1)^, call the cuckoo* i*, a Levy flight can be defined as in the following:

(8) $$\begin{array}{c} x_{i}^{\left(t+1\right)}=x_{i}^{\left(t\right)}+\alpha ⊕\text{Levy}\left(\lambda \right), \end{array}$$

where *α* > 0 is the step size which should be connected with the solution space. In general, we can set *α* = *O*(1). In essence, (8) is a stochastic equation for random walk, which is a Markov chain whose next location only relies on the current location and the transition probability. The product *⊕* means entry-wise multiplications. This entry-wise product is similar to those used in PSO, the random walk via Levy flight is more efficient in searching the solve space, and its step length is much longer in the long run.

In essence, the Levy flight provides a random walk; at the same time, the random step length is drawn from a Levy distribution, which has an infinite variance with an infinite mean:

(9) $$\begin{array}{c} \text{Levy}\sim u=t^{-\lambda }\;\;\;\left(1<\lambda \leq 3\right). \end{array}$$

Here, the consecutive steps of a cuckoo essentially constitute a random walk process which obeys a power-law step-length distribution with a heavy tail. Some of the new solutions should be generated by Levy flight around the best solution; this will accelerate the local search. However, a large proportion of the new solutions may be generated by extensive randomization, whose locations may be far from the current best solution; this will make sure the algorithm will not fall into a local optimum.

### 3.2. CS-PSO Algorithm

The parameters *p*_*a*_, *λ*, and *α* introduced in the CS help the algorithm to find optimal solution. Among them, *p*_*a*_ is a very important parameter in determining the proportion of worse nests and can be potentially used in adjusting convergence rate of algorithm. The traditional CS algorithm uses a fixed value for *p*_*a*_. This value is set in the initialization stage and cannot be changed during the whole iterative processes. The main drawback of this method is that it is not very easy to find the best proportion. The proportion of worse nests too big or too small will all lead to a case that the algorithm cannot obtain the optimal solution.

Particle swarm optimization (PSO) [25] is an optimization algorithm proposed by Eberhart and Kennedy in 1995. It is a stochastic optimization algorithm of swarm intelligence based on the simulation of various collective behaviors of the living creatures such as bird flocking, fish schooling, and swarm theory. As an optimization tool, PSO provides a population-based search strategy in which individuals are called particles. In PSO, particles fly around in a multidimensional search space. All of the particles are evaluated by the objective function and have a certain velocity which influences the movement of particles. The velocity and position vector is updated by the following equations:

(10)   vit+1=Wt∗vit+c1∗r1∗p_bestit−xit+c2∗r2∗g_bestt−xit

(11) $$\begin{array}{c} x_{i}^{t+1}=x_{i}^{t}+v_{i}^{t+1} \end{array}$$

(12) $$\begin{array}{c} W^{t}=\frac{M_{\text{gen}}-t}{M_{\text{gen}}}, \end{array}$$

where *x*_*i*_^*t*^ and *v*_*i*_^*t*^ signify the position and velocity of particle at time *t*, *W* ^*t*^ is inertia weight, *M*_gen_ is the maximum generations of the algorithm, *c*_1_ and *c*_2_ are positive acceleration constants, *r*_1_ and *r*_2_ are random values generated in the range [0,1], sampled from a uniform distribution, *p*_*best*_*i*_ is the best solution of *i*th individual particle, and *g*_*best* is the best solution tracked by any particle among all generations of the swarm.

CS-PSO utilizes PSO algorithm as a disturbance, substitute for the process of updating the worse nests in CS algorithm. *p*_*best* and *g*_*best* enable the PSO algorithm to effectively develop the local solutions into global optimum solutions. The disturbance has nothing to do with the worse nests, which makes a broader hunting and rapidly converges to the optimal solution.

## 4. Proposed Methodology

In order to obtain the enhanced image, a transformation function defined in (1) is used. The function contains two parameters, namely, *α* and *β*, as stated in Section 2; *α* and *β* exert a considerable influence on the performance of image enhancement. The main idea of applying CS-PSO to search the best parameters pair (*α* and *β*) is as follows.

Each position vector of the CS-PSO stands for a candidate parameters pair for *α* and *β*. The initial population is generated with *N* number of solutions randomly within their range and corresponding random velocities and each solution is a *D*-dimension vector, here *D* is set as 2 that each solution represents 2D candidate parameters. *X*_*i*_ represents the *i*th bird position in the population which denotes a candidate parameter pair and its fitness can be measured by fitness function defined in (6). After calculating all of the fitness values, *p*_*best* and *g*_*best* can be obtained. In CS each individual is generated by the equation defined in (8). Then, in PSO each particle is disturbed to the direction of best solution as it is reflected in (10) and (11). With the defined movement rules, the algorithm will run until it terminates and outputs the best position as the optimal parameters for *α* and *β*. In all, the basic procedures of image enhancement by using CS-PSO can be depicted with the pseudocode as follows in Pseudocode 2.

## 5. Simulation Results and Discussion

In this section, in order to make a comparison for optimization ability, image enhancement technology based on incomplete Beta function has been studied formerly by the authors using some traditional optimization algorithms like GA and PSO, and some newly proposed evaluation algorithms like DSA, BSA, and basic CS. Therefore, comprehensive comparisons are provided between the optimum solutions obtained for these problems using the proposed CS-PSO algorithm and other metaheuristic algorithms.

For a fair comparison of results, the search process is terminated before the maximum number of iterations is attained. The main parameters used for these approaches are as follows: the population for all the algorithms is the same; that is 50, and all these algorithms will terminate after being executed 100 times. Moreover, the selection rate for GA is 0.9, the crossover rate is 0.8, and mutation rate is 0.1. For PSO, the cognitive coefficient *C*_1_ = 2.0 and *C*_2_ = 2.0; the value of inertia weight is set as 1. For CS, the control parameter Pa = 0.25. For DSA, the control parameter *p*_1_ = 0.3 and *p*_2_ = 0.3. And for BSA, the special parameter of mix rate is fixed to 1. In the paper, there are two problem specific parameters *α* and *β*. The range of these parameters is the same as [26]. *α* ∈ [0,10] and *β* ∈ [0,10].

All of the algorithms are programmed and implemented with Matlab R2012b on a personal computer with 2.53 GHz CPU, 2G RAM running memory in windows XP system. In the paper, four images are used to evaluate the enhancement technique based on CS-PSO. The general information of these four images is shown in Table 1. In order to show the optimization ability and enhancement quality of CS-PSO, results of the proposed method are compared with five other methods, namely, (i) linear contrast stretching (LCS), (ii) histogram equalization (HE), (iii) BSA based image enhancement (BSAIE), (iv) bijective DSA based image enhancement (DSAIE), (v) GA based image enhancement (GAIE), (vi) PSO based image enhancement (PSOIE), and (vii) CS based image enhancement (CSIE). All the algorithms are evaluated using the same objective function which is proposed in the paper, and the results are put in Table 2. On the other hand, in order to show the good quality of the objective function, results of the proposed method are compared with Apurba's method [12], Zhou's method [27], and Eskicioglu's method [28], and the results are given in Tables 3 and 4.

In Table 1, Min-pixel and Max-pixel in last column signify the min and max intensity values of pixel in the original image. BV and WV in Table 2, respectively, indicate the best and worst fitness value of objective function and STD is the variance of the fitness value by making 100 independent operations. In Table 4, the unit of time is the second.

### 5.1. Comparison of Optimization Ability

Table 2 shows the qualities of the test images which are enhanced using the above mentioned methods and the proposed objective functions are measured in terms of BV and WV; it is obvious that the enhancement technique based on evolutionary algorithm produces better results than LCS method and HE method according to the fitness value of objective function. In the aspect of optimization ability, GAIE, PSOIE, and BSAIE method are apparently worse than DSA, CS, and CS-PSO based image enhancement methods; DSAIE method can obtain good solution in general, but the variance of the fitness value in average of 100 independence experiments is obviously more than CS and CS-PSO; it reflects that the stability of the above four methods is not very well. CSIE method can converge to better solution, the variance of the fitness value in average of 100 independence experiments is less than DSAIE, BSAIE, GAIE, and PSOIE, and it can converge to the optimal solution for I3 image; the fitness value is slightly more than the proposed method, but for other images, the fitness value is equal or inferior to the proposed method; in addition, as for the value of WV and the variance of the fitness value in average of 100 independence experiments is also worse than the proposed method for all images. As a result, compared with the above methods, the proposed method is relatively more stable. More importantly, it can converge to the optimal solution as quickly as possible.

### 5.2. Comparison of Objective Function

As could be observed from Table 2, the CS-PSO has the best optimization ability in these six algorithms. To test the proposed measure for image enhancement, CS-PSO is run on these four objective functions for image enhancement, and LCS and HE are also used to make a comparison for these four objective functions. The fitness values of Apurba's method, Zhou's method, Eskicioglu's method, and the proposed method optimized by CS-PSO are given in Table 3, and running time of each method is listed in Table 4. Seen from Table 3, for most of test images, Eskicioglu and Zhou's method combined with CS-PSO provide less or approximate fitness value compared with LCS and HE method, which shows that the performance of Eskicioglu and Zhou's method is inferior to LCS and HE method as a whole. Apurba's method optimized with CS-PSO could produce better fitness value than LCS and HE method; it may be used for evaluating the quality of the enhancement technique under certain conditions. Table 4 shows that Zhou's objective function has the shortest running time among these methods. However, it cannot assure the quality of enhanced image. Although Apurba's objective function can obtain acceptable result, its running time is much higher than other three objective functions, which will bring down the efficiency of image enhancement. So, compared with the above three objective functions, the proposed objective function can achieve a better balance between image quality and computing time, which is more feasible in practical image enhancement.

### 5.3. Visual Evaluation of the Enhanced Image

**Figure 2:**
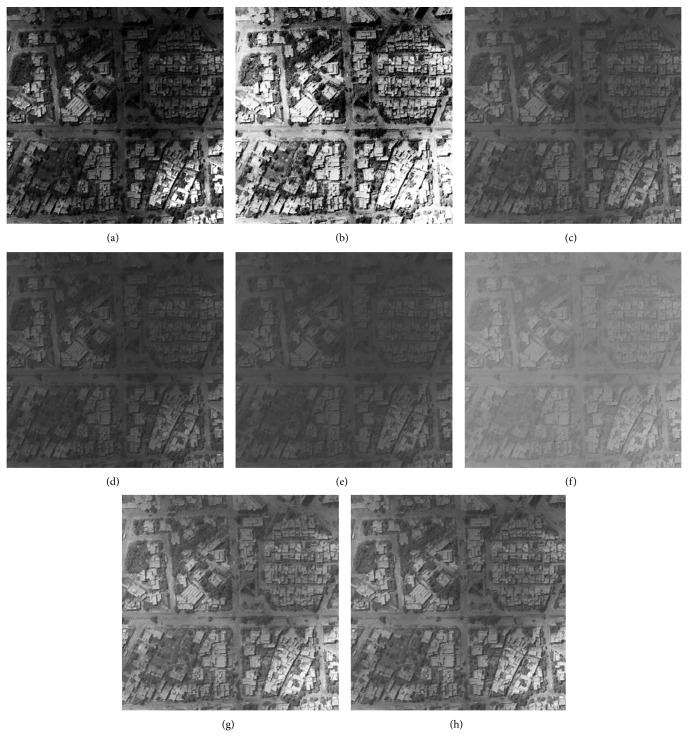
Enhanced result of I1 image: (a) LCS, (b) HE, (c) DSA, (d) BSA, (e) GA, (f) PSO, (g) CS, and (h) CS-PSO.

**Figure 3:**
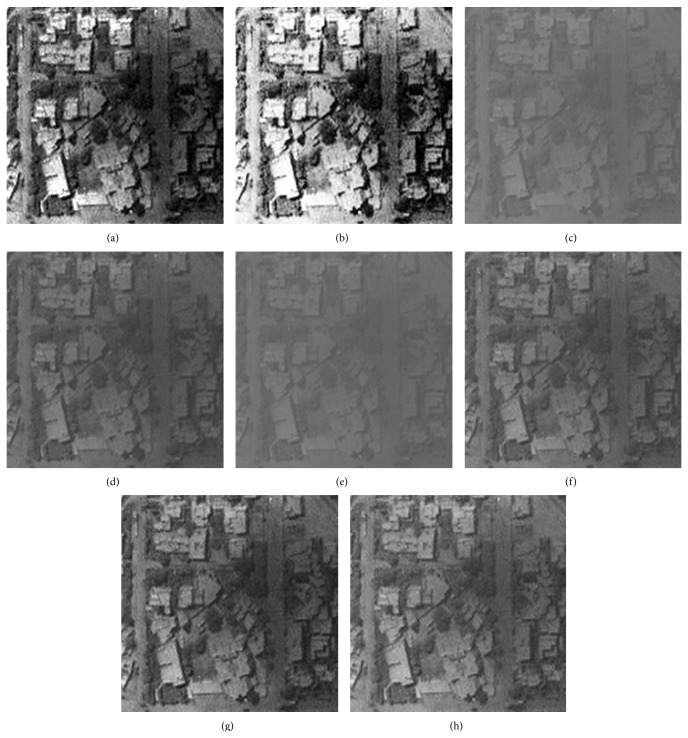
Enhanced result of I2 image: (a) LCS, (b) HE, (c) DSA, (d) BSA, (e) GA, (f) PSO, (g) CS, and (h) CS-PSO.

**Figure 4:**
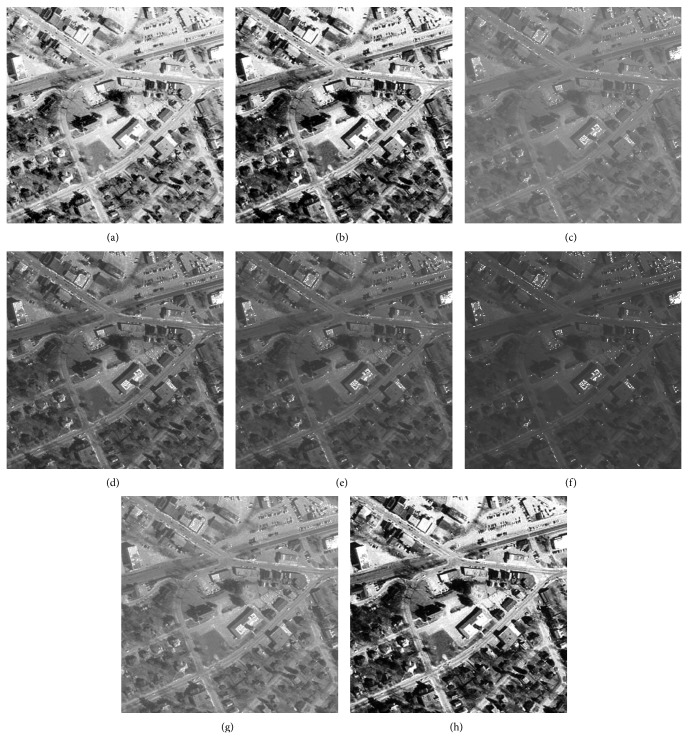
Enhanced result of I3 image: (a) LCS, (b) HE, (c) DSA, (d) BSA, (e) GA, (f) PSO, (g) CS, and (h) CS-PSO.

**Figure 5:**
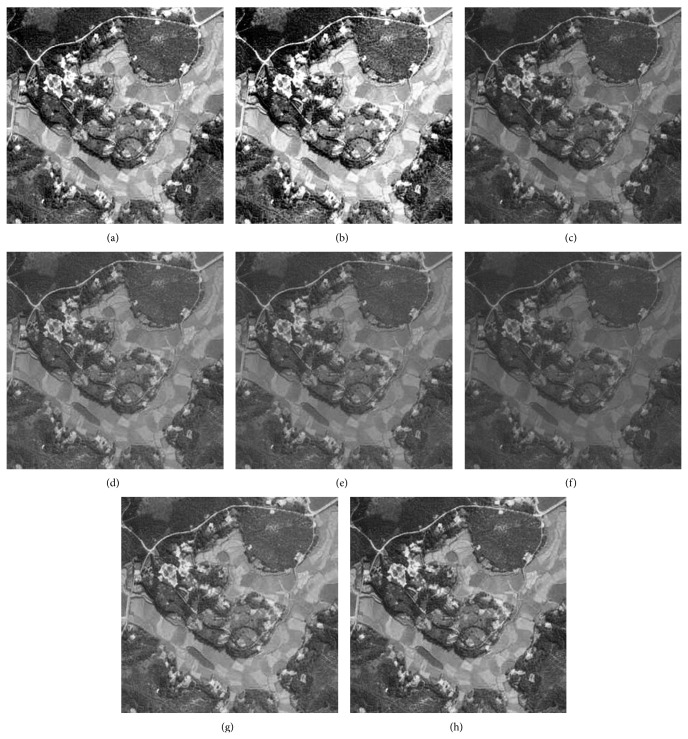
Enhanced result of I4 image: (a) LCS, (b) HE, (c) DSA, (d) BSA, (e) GA, (f) PSO, (g) CS, and (h) CS-PSO.

The qualitative performance of CPIE and the contemporary methods are illustrated using four images which are given in Figures 2, 3, 4, and 5. The enhanced images of the same by LCS, HE, GAIE, PSOIE, CSIE, and CPIE are shown in Figures 2, 3, 4(a)–4(h) and 5(a)–5(h) respectively. In Figures 2(a)-2(b), the enhanced image is too bright. As the enhancement for the entire image, the noise portions are also enhanced. In Figures 2(c)–2(f), the detail portions in the enhancement image clearly show the brightness degradation and overenhancement; the detail section is not clearly visible in the dark region. The same abrupt brightness change can be notified in other images (Figures 3, 4, and 5(c)–5(f)) also. Though there is not much brightness change in the results of CSIE (Figures 2, 3, 4, and 5(g)), the low frequency portions of them are not found to be enhanced at all.

Figures 2, 3, 4, and 5(h) are the visual results of the proposed method which are better than those of other image enhancement techniques and are free from brightness change and overenhancement. The detail sections are clearly visible in the dark region. The foreground, background, and the target can be clearly distinguished; the noise is effectively rejected. Most of the frequency ingredient obtains prominent enhancement. At the same time, the proposed method has been found to produce comparatively better results for the other test images too.

## 6. Conclusion

In summary, a CS-PSO based image enhancement technique for gray-level images is proposed and a novel criterion for measuring quality of the enhanced image is given in the paper. Results of the proposed method are compared with some other image enhancement techniques, like linear contrast stretching, histogram equalization, and incomplete Beta function based image enhancement method optimized with Bijective DSA, BSA, GA, PSO, and CS. It is observed that evolutionary algorithm can be well used in image enhancement according to the quality of enhanced image. Meanwhile, objective function plays a decisive role in evaluating the enhanced image. Among these methods, our method can quickly and stably converge to the optimal solution and the objective function is also better than other methods.

## Figures and Tables

**Pseudocode 1:**
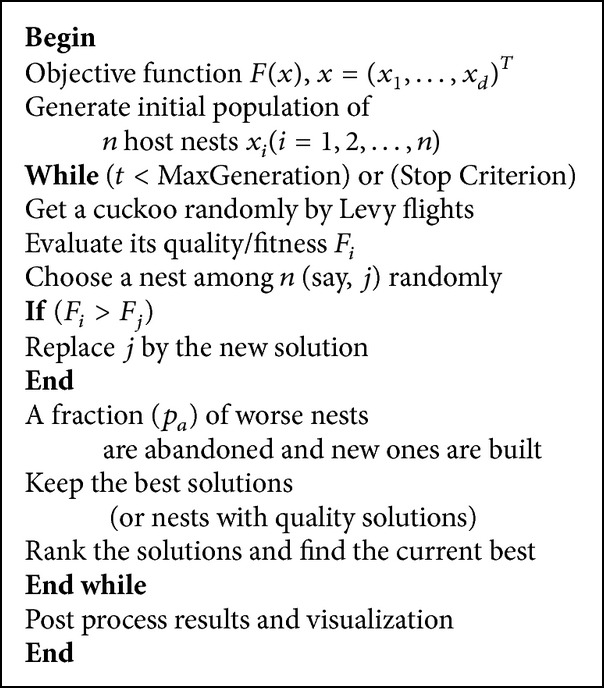
Pseudocode of cuckoo search via Levy flights.

**Pseudocode 2:**
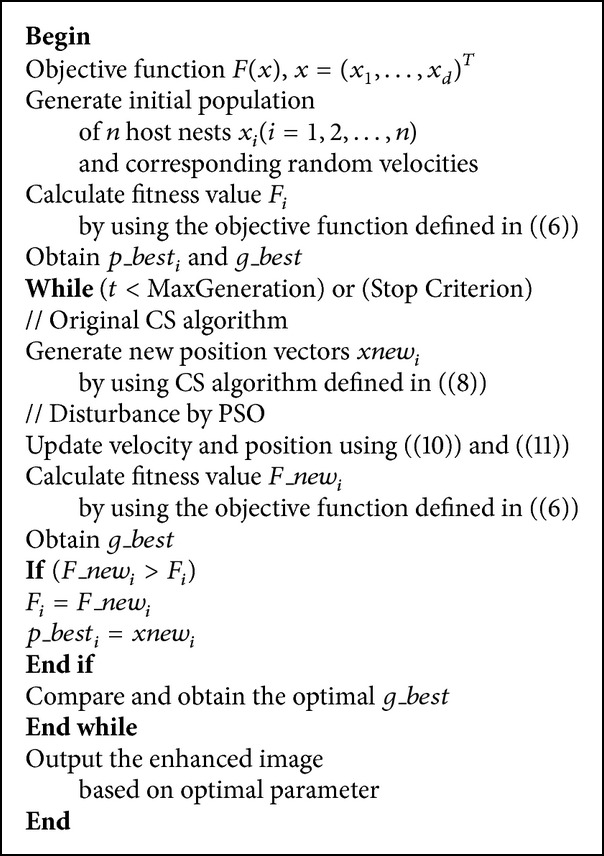
The pseudocode of CS-PSO based image enhancement.

**Table 1:** Basic information of four test images.

Image	Size	Min-pixel	Max-pixel
I1	328 ∗ 358	0	202
I2	170 ∗ 154	7	70
I3	364 ∗ 366	101	255
I4	325 ∗ 308	69	239

**Table 2:** Fitness value of all the methods by 100 independent operations.

Image	Meas.	LCS	HE	DSA	BSA	GAIE	PSOIE	CSIE	Proposed
I1	BV	3.4424	3.6439	8.8374	8.8321	8.7551	8.8358	8.8681	8.8681
	WV	3.4424	3.6439	8.7280	8.4802	8.0349	8.5353	8.7968	8.8318
	STD.	—	—	0.0398	0.1263	0.2828	0.1039	0.0232	0.0139

I2	BV	4.7464	3.4810	8.4899	8.4548	8.5182	8.5181	8.5194	8.5198
	WV	4.7464	3.4810	8.3842	8.3045	7.7686	8.2877	8.4416	8.4743
	STD.	—	—	0.0365	0.0460	0.2407	0.0873	0.0306	0.0190

I3	BV	4.0961	3.9274	10.2577	10.0611	10.2690	10.2290	10.2762	10.2721
	WV	4.0961	3.9274	9.9720	9.4963	9.6413	9.9563	10.0900	10.1958
	STD.	—	—	0.0876	0.2153	0.2122	0.0906	0.0611	0.0482

I4	BV	5.6280	4.0179	10.4347	10.3822	10.1717	10.4581	10.4584	10.5047
	WV	5.6280	4.0179	10.1303	9.6348	9.3446	10.2372	10.3745	10.4354
	STD.	—	—	0.1064	0.2643	0.3106	0.1185	0.0331	0.0285

**Table 3:** Fitness value by using proposed and other three objective functions.

Objective function	Method	I1	I2	I3	I4
Apurpa	LCS	0.3321	0.3431	0.4849	0.4216
	HE	0.3015	0.2598	0.4292	0.3664
	CS-PSO	0.5147	0.4667	0.5673	0.5987

Zhou	LCS	0.0864	0.0868	0.0861	0.0859
	HE	0.0325	0.0503	0.0593	0.0574
	CS-PSO	0.0337	0.0180	0.0925	0.0833

Eskicioglu	LCS	0.0657	0.1829	0.0691	0.0720
	HE	0.1178	0.2414	0.0852	0.0886
	CS-PSO	0.0677	0.1194	0.0885	0.0847

Proposed	LCS	0.3442	0.47464	0.4096	0.5628
	HE	0.3643	0.34810	0.3927	0.4017
	CS-PSO	0.8868	0.8519	1.0272	1.0504

**Table 4:** Running time of each iteration compared with other objective functions.

Image	Apurba	Zhou	Eskicioglu	Proposed
I1	43.15	0.050	6.73	0.19
I2	8.73	0.023	0.72	0.15
I3	46.80	0.052	6.57	0.19
I4	36.84	0.060	6.48	0.20
